# Assessment of nutritional status in children with kidney diseases—clinical practice recommendations from the Pediatric Renal Nutrition Taskforce

**DOI:** 10.1007/s00467-020-04852-5

**Published:** 2020-12-14

**Authors:** Christina L. Nelms, Vanessa Shaw, Larry A. Greenbaum, Caroline Anderson, An Desloovere, Dieter Haffner, Michiel J. S. Oosterveld, Fabio Paglialonga, Nonnie Polderman, Leila Qizalbash, Lesley Rees, José Renken-Terhaerdt, Jetta Tuokkola, Johan Vande Walle, Rukshana Shroff, Bradley A. Warady

**Affiliations:** 1 University of Nebraska, Kearney, NE USA; 2 University College London Great Ormond Street Hospital Institute of Child Health, London, WC1N 3JH UK; 3 University of Plymouth, Plymouth, UK; 4 Emory University, Atlanta, GA USA; 5 Children’s Healthcare of Atlanta, Atlanta, GA USA; 6 University Hospital Southampton NHS Foundation Trust, Southampton, UK; 7 University Hospital Ghent, Ghent, Belgium; 8 Children’s Hospital, Hannover Medical School, Hannover, Germany; 9 Emma Children’s Hospital, Amsterdam University Medical Center, Amsterdam, The Netherlands; 10 Fondazione IRCCS Ca’ Granda Ospedale Maggiore Policlinico, Milan, Italy; 11 British Columbia Children’s Hospital, Vancouver, Canada; 12 Great Northern Children’s Hospital, Newcastle upon Tyne, UK; 13 Wilhelmina Children’s Hospital, University Medical Center Utrecht, Utrecht, The Netherlands; 14 Children’s Hospital and Clinical Nutrition Unit, Internal Medicine and Rehabilitation, University of Helsinki and Helsinki University Hospital, Helsinki, Finland; 15 Children’s Mercy Kansas City, Kansas City, MO USA

**Keywords:** Assessment, Children, Kidney diseases, Nutrition, Clinical practice recommendations, Pediatric Renal Nutrition Taskforce

## Abstract

In children with kidney diseases, an assessment of the child’s growth and nutritional status is important to guide the dietary prescription. No single metric can comprehensively describe the nutrition status; therefore, a series of indices and tools are required for evaluation. The Pediatric Renal Nutrition Taskforce (PRNT) is an international team of pediatric renal dietitians and pediatric nephrologists who develop clinical practice recommendations (CPRs) for the nutritional management of children with kidney diseases. Herein, we present CPRs for nutritional assessment, including measurement of anthropometric and biochemical parameters and evaluation of dietary intake. The statements have been graded using the American Academy of Pediatrics grading matrix. Statements with a low grade or those that are opinion-based must be carefully considered and adapted to individual patient needs based on the clinical judgment of the treating physician and dietitian. Audit and research recommendations are provided. The CPRs will be periodically audited and updated by the PRNT.

**Supplementary Information:**

The online version contains supplementary material available at 10.1007/s00467-020-04852-5.

## Introduction

The nutritional assessment encompasses both the presenting nutritional status and consequent dietary intervention recommendations. In children with kidney diseases, a comprehensive assessment requires multiple evaluations including dietary evaluation and anthropometric and biochemical measurements. In this document, the Pediatric Renal Nutrition Taskforce (PRNT) addresses the assessment of nutritional status in children with kidney diseases.

The PRNT performed an extensive literature review and considered all relevant studies in the development of these CPRs. Where there is a paucity of literature on this topic, expert opinion has been utilized when evidence was inadequate. These CPRs provide a structure for decision-making but may need to be adapted by the clinician for the individual patient using clinical judgment. Whenever possible, nutritional assessment should be completed by a trained pediatric renal dietitian.

## Methods

The guideline development process, including the group composition and task distribution, is described in previous PRNT publications [1].

### Developing the PICO questions

Clinical practice recommendations are most useful when they provide well-defined actionable advice on choosing between alternative approaches in specific clinical situations. We developed clinical questions to be addressed by each statement and framed them in a searchable format, with specification of the patient group (P) to whom the statement would apply, the intervention (I) being considered, the comparator (C) (which may be “no action” or an alternative intervention), and the outcomes (O) affected by the intervention. Our PICO terms were as follows:

- Population: children from birth to 18 years of age with kidney diseases
- Intervention: assessment of nutritional requirements
- Comparator: assessment in healthy age- and sex-matched pediatric populations or no comparator
- Outcomes: assessment of growth (including underweight, overweight, obesity, and malnutrition), energy, and protein requirements and adequacy of the nutritional intake

### Literature search

An electronic search using PubMed and an inclusive academic library search (including MEDLINE, Cochrane, and Scopus databases) was performed using the search terms and strategy detailed in Supplementary Table 1. Limits were preset to only include manuscripts published in the English language between 1980 and 2018. Given the paucity of studies in this field, all publications, including meta-analyses, prospective observational studies (irrespective of patient numbers), and retrospective observational studies (with more than 20 children), have been included.

### Framing advice

The previously published CPR for the dietary management of calcium and phosphate [1] has outlined the development process and purpose of the recommendations. Statements have been graded using the American Academy of Pediatrics grading matrix (Supplementary Table 2) and were submitted to a Delphi procedure, as previously described [1], to validate expert opinion.

## Clinical practice recommendations

1. Anthropometric assessment
  1.1 Measure weight, height, or length and head circumference in children with kidney diseases (grade A, strong recommendation)
    1.1.1 Use euvolemic (dry) weight for nutritional assessment, with adjustment of measured weight when indicated (e.g., being on dialysis and having nephrotic syndrome) (grade A, strong recommendation)
    1.1.2 Measure recumbent length under 2 years of age and standing height thereafter. When young children are unable to stand for an accurate height measurement, recumbent length can be measured (grade A, strong recommendation)
      1.1.2.1 Use a surrogate measurement of height for older children who are unable to stand (grade D, weak recommendation)
    1.1.3 Measure the head circumference in all children up to 2 years of age or up to 3 years of age when appropriate centile charts are available (grade A, strong recommendation)
  1.2 Plot anthropometric measurements serially on centile growth charts. Use the World Health Organization (WHO) growth chart for all ages or country-specific growth charts, if available, beyond 2 years of age (grade A, strong recommendation)
    1.2.1 Calculate *z*-scores (standard deviation scores (SDS)) to complement growth chart plots (ungraded, expert opinion)
    1.2.2 Calculate height/length velocity *z*-scores over a minimum period of six months (grade B, moderate recommendation)
    1.2.3 Use disorder- or genetic condition-specific growth charts when applicable (B, moderate recommendation)
    1.2.4 Utilize trends in growth parameters to assist clinical decision-making (grade D, weak recommendation)
  1.3 Calculate body mass index (BMI) in children aged 2 years and older and weight for length in children younger than age 2 (A, strong recommendation)
    1.3.1 Plot BMI or weight for length on centile growth charts (grade B, moderate recommendation)
    1.3.2 Calculate BMI or weight-for-length *z*-scores/SDS to complement growth chart plots (grade B, moderate recommendation)
    1.3.3 Use height age for determining BMI *z*-score/SDS if the child is shorter than the third centile curve on the growth chart, provided the child has not reached their adult height (grade B, moderate recommendation)
  1.4 Calculate midparental height and plot the value as a centile to estimate growth potential (grade C, weak recommendation)
  1.5 For premature infants, plot weight, length, and weight for length for both gestational and chronological age for the first year of life if born from 32 up to 37 weeks of gestation and through 2 years of age if born prior to 32 weeks of gestation (grade D, weak recommendation)
  1.6 Monitor growth parameters routinely in children with kidney diseases, with increased frequency in younger children, and in those children with advanced CKD, with comorbidities, and with risk factors for poor growth and those not meeting nutritional and growth targets (grade D, weak recommendation)

### Evidence and rationale

#### Euvolemic weight

An important consideration for the nutritional assessment of children with kidney diseases, especially those on dialysis, is the use of euvolemic or “dry” weight. Euvolemic weight is a child’s true weight, and without identifying the euvolemic weight, miscalculations and errors may take place in patient management. Clinicians should make every attempt to determine euvolemic weight or to estimate it by the best means possible. Many children with kidney diseases, especially those with oliguria or anuria or those with active nephrotic syndrome, retain fluid, and thus, their measured weight exceeds their euvolemic weight. For children who receive dialysis, the aimed-for weight at the end of a hemodialysis (HD) session or after overnight peritoneal dialysis (PD) for those patients who receive either continuous ambulatory PD (CAPD) or automated PD (APD) should be utilized, recognizing that this measurement may still not reflect the euvolemic weight. In patients who receive APD with a last fill or in those who receive CAPD, the volume of the instilled dialysis solution should be subtracted from the measured weight. Indicators of excess weight due to fluid include edema on physical examination and hypertension that is responsive to fluid removal during dialysis. Children with high urine and sodium losses may actually fall below their euvolemic weight if dehydrated, and this should be considered when carrying out the assessment [2]. The recommended frequency of evaluation of euvolemic weight is outlined in Table 1.

**Table 1:** Parameters and frequency of nutritional assessment in children with CKD stages 3b–5D^#^. Anthropometric measurements

Measure	Age 0–1 year^∞^ Minimum interval (weeks)		Age 1–3 years Minimum interval (months)		Age > 3 years Minimum interval (months)	
	CKD 3b–5	CKD 5D	CKD 3b–5	CKD 5D	CKD 3b–5	CKD 5D
Height or length for age (centile or SDS)	6	2–4	2	1	3	3
Height or length (centile or SDS)	8	4	3	2	6	6
Height velocity for age (SDS)	N/A	N/A	3	2	6	6
Estimated euvolemic weight and weight for age (centile or SDS)	6	4	2	1	3	3
BMI for height age (centile or SDS)	N/A	N/A	2*	1*	3	3
Weight for length* (centile or SDS)	6	6	2*	1*	N/A	N/A
Head circumference for age (centile or SDS)	6	4	2	2	N/A	N/A

^#^Earlier stages of CKD and other kidney diseases are not addressed in this table, as clinical conditions can vary and physician discretion is required. Further details are addressed in [2]

*Weight for length should be used for children < 2 years of age or up to 3 years if accurate standing height measurement is not possible

^∞^Infants and toddlers cannot be categorized with the stage of CKD as there may be spontaneous improvement in kidney function up to 2 years of age. A suggested method to characterize the stage of CKD in this age group is to use the KDIGO 2012 clinical practice guideline for the evaluation and management of chronic kidney disease, substituting a GFR > 1 but ≤ 2 SDS below the mean for moderate reduced GFR (stages 3–4) and severely reduced GFR > 2 SDS below the mean for stage 5 [3]

**Table 2:** Expected daily weight gain for age (g)

Age/parameter	Daily weight gain (g/kg/d)
Premature, currently < 2 kg*	15–20
Premature, currently > 2 kg*	20–30
0–4 months	23–34
4–8 months	10–16
8–12 months	6–11
12–16 months	5–9
16–24 months	4–9

*Use only until reaching term gestational age of 37 weeks

Adapted from Beer et al. [41], used with permission

**Table 4:** Parameters and frequency of nutritional assessment in children with CKD stages 3b–5D^#^. Dietary intake

Age 0–1 year^∞^ Minimum interval (weeks)		Age 1–3 years Minimum interval (months)		Age > 3 years Minimum interval (months)	
CKD 3b–5	CKD 5D	CKD 3b–5	CKD 5D	CKD 3b–5	CKD 5D
8	8	3	3	6	4

^#^Earlier stages of CKD and other kidney diseases are not addressed in this table, as clinical conditions can vary and physician discretion is required. Further details are addressed in [2]

^∞^Infants and toddlers cannot be categorized with the stage of CKD as there may be spontaneous improvement in kidney function up to 2 years of age. A suggested method to characterize the stage of CKD in this age group is to use the KDIGO 2012 clinical practice guideline for the evaluation and management of chronic kidney disease, substituting a GFR > 1 but ≤ 2 SDS below the mean for moderate reduced GFR (stages 3–4) and severely reduced GFR > 2 SDS below the mean for stage 5 [3]

#### Linear growth

Children with chronic kidney disease (CKD) may have poor linear growth, which is the result of a number of factors, including metabolic acidosis, inadequate nutrition, renal osteodystrophy, sodium depletion, delayed sexual maturation, and abnormalities of the growth hormone insulin-like growth factor 1 axis [2, 4–6]. Many of these same factors also affect weight gain. Children with other kidney diseases, such as renal tubular acidosis, may also experience growth failure [7]. Length (before 2 years of age) or height (from 2 years of age) should be regularly measured by trained personnel, ideally the same individual, serially and compared with age- and sex-specific reference charts for healthy children [2]. The recommended frequency of measurement is outlined in Table 1. Gestational age should be taken into account when assessing the length of children born prematurely. Pubertal status and bone age should also be considered when assessing linear growth. Evaluation for pubertal delay can help determine whether alternative measurements, such as height age and BMI calculated for height age, are appropriate considerations for the patient assessment and if there is an opportunity for catch-up linear growth. In the absence of information about pubertal status, height age may act as a surrogate of physical development. While this is also important when assessing the potential need for recombinant growth hormone therapy, this issue is outside the scope of this document. Recently published recommendations from the European Society for Paediatric Nephrology address the use of growth hormone in children with CKD [8].

Standardized techniques for measurement of recumbent length or standing height should be used, which include maintaining the subject’s eyes and ears parallel for the measurement, removal of hair accessories or head pieces, and placement of the heels, buttocks, and shoulders of the subject flat against the length board or stadiometer. Recumbent length is useful to evaluate children who are unable to stand and is recommended for children under the age of 2–3 years, depending on their ability to stand erect. Two persons are needed to accurately obtain a recumbent length and to help hold the child in the correct position [2]. While this approach to recumbent length measurement may also be necessary in older children who are unable to stand, it is less reliable in older and taller children. Recumbent length overestimates height by an average of 0.7 cm with greater variation likely in taller children [9]. Although there are no studies in children with kidney diseases, arm span appears to be the most useful surrogate measurement for height in older children who are unable to stand, although it may slightly underestimate standing height. Several studies indicate that arm span is highly correlated with measured height. Even though it typically slightly underestimates height, it is highly reproducible and consistent, providing a valuable tool for trending growth velocity [10–12]. Demi-span is a practical tool to simplify this process. Demi-span is a measurement from the midline of the body parallel to the tip of the third finger; when doubled, it provides the total arm span. Serial measurements are more useful than single measurements for evaluation of linear growth with surrogate tools, given the potential for variation from the actual height. In addition, the use of the same surrogate when measuring serially is important for accuracy in trending because each surrogate has its own individual limitations in terms of under- or overestimation. For children with contractures or joint deformities, ulnar length may be the most useful surrogate, although it may overestimate standing height. The use of calipers optimizes accuracy of this measurement, but a simple tape measure provides nearly accurate results [13, 14]. Other surrogate measurements are available, depending on patient circumstances, that the clinician may use to obtain the most accurate estimate of height [12, 13]. Equations based on the use of ulnar length and arm span are included in the supplementary material.

#### Head circumference

A small head circumference may indicate chronic inadequate nutrition in the absence of other comorbidities that explain the small head circumference. Conditions that cause macrocephaly (e.g., hydrocephalus) limit the value of head circumference as a marker of nutritional status [2]. Warady et al. described a cohort of young peritoneal dialysis patients, the majority of whom demonstrated appropriate cognitive development and normal head circumferences. It is noteworthy to recognize the difference between these patients and previous reports of children with CKD and small head circumferences which was the provision of improved nutrition and adequate dialysis and the absence of aluminum-based binders in the former patients [15]. The recommended frequency of head circumference evaluation is included in Table 1.

#### Tracking growth

Plotting growth on standard growth charts remains the best way to visualize growth trends and determine if a child is crossing growth centiles. The WHO growth charts are the worldwide standard for tracking growth in children younger than the age of 2 years because they represent the growth achieved by a healthy, breastfed, and immunized population. Major health organizations such as the Centers for Disease Control support the use of the WHO growth charts in children under the age of 2. After the age of 2, if available and validated, up-to-date growth charts that are country or region specific should be used since growth can vary based on ethnicity. In their absence, the WHO growth charts remain a good option. The determination of standard deviation scores (SDS) or *z*-scores is an excellent complementary tool to use alongside growth charts. The extent to which a child is outside the range of the 3rd to the 97th centiles is most accurately identified with the use of *z*-scores. The equation used to determine SDS is noted below, and many validated SDS calculators are available online. Height and length velocity SDS scores require 6 months of data, ideally, to accurately characterize a trend in the growth data [2, 9].

Comorbidities should be considered when assessing length or height in children with other medical conditions such as cerebral palsy or spina bifida that may alter expected linear growth. Only conditions in which altered growth is a known part of the physiology of the disorder should be evaluated with different growth charts, as opposed to conditions in which growth may be altered by poor care or comorbid conditions related to the primary condition. Condition-specific growth charts should be used for children with genetic or metabolic disorders in which growth potential may be altered (e.g., trisomy 21 (Down syndrome), Wolf–Hirschhorn syndrome, and Prader–Willi syndrome) [16–19]. As is the case with healthy children, following growth trends is important when evaluating children with these conditions to best assess growth progress.

#### BMI and body composition

Children with CKD have increased fat-to-muscle ratios and decreased lean mass. BMI does not allow for distinction between muscle mass and fat mass [20–23]. Muscle deficits are common because of lean mass wasting [21]. This phenotype typically persists post-kidney transplant [23–27]. This suggests that even children with CKD and a normal BMI may have excess adiposity. The latter is especially true as CKD progresses [26]. These imbalances may be present even with adequate physical activity [21]. BMI may also be misleading because many children with CKD have growth retardation [27]. Consequently, short children with CKD may have a normal weight compared with pediatric norms, but their short stature leads to a raised BMI [4]. Although for any centile below an SDS of 0 it has been proposed that correction should be considered, this taskforce has chosen the 3rd centile (−1.88 SDS) as a reasonable cut-off. In fact, BMI adjustments for height age (age at which an individual’s height would be at the 50th centile) have been validated in the literature as being more accurate than the unadjusted BMI for children < 3rd centile in height. The use of BMI for actual age in short children may overestimate the incidence of underweight and therefore lean mass, whereas the use of BMI for height age may more accurately reflect true lean mass or adiposity [27]. In the peripubertal or pubertal periods, height age is also considered to be a good surrogate of physical development. However, this adjustment is inappropriate once a child has attained final adult height [2].

Despite the influential factors noted above, BMI, or weight for length for children younger than 2 years, remains the best primary measure of malnutrition or adiposity for children with kidney disease. It is easy to calculate and is indicative of overweight or obesity at the upper end and of lean muscle wasting or underweight at the lower end. Plotting BMI on growth charts and tracking centiles provide valuable information that can be evaluated alongside clinical judgment to assess the adequacy or excess of weight gains and proportionality. Children on dialysis with either very high or low BMI values are at greater risk for mortality [28], although the mechanism for this increased risk is unknown. The recommended frequency of BMI determination is outlined in Table 1.

BMI calculation:

Weight in kilograms/height in m^2^

BMI typically rapidly increases in children who have undergone kidney transplantation. In the first 18 months post transplant, obesity rates double with the most significant changes in weight taking place in the first 6 months [29, 30]. Nevertheless, malnutrition remains prevalent in a subset of transplant recipients, especially those with medication-related gastrointestinal (GI) side effects or low BMI prior to transplant [31]. Children receiving long-term steroid therapy, such as those with nephrotic syndrome, may experience weight gain and excess adiposity [32]. Conversely, failure to thrive occurs in children with a normal GFR who suffer from excessive tubular losses of salt and water, as well as in those with uncorrected renal tubular acidosis [2, 33]. Hence, BMI and weight for length are important evaluation tools to identify children with inadequate or excessive energy intake.

#### Midparental height

Midparental height is calculated to assess a child’s linear growth potential [2, 34]. For the best accuracy, measuring the heights of both the biological mother and father is ideal [35]. A standard deviation of 8.5 cm in the calculation is the expected variation in achieved height; thus, the majority of healthy children will be within 8.5 cm of their predicted final adult height. A child’s current height centile should be compared with the height centile of the child’s calculated midparental height at age 18–20 years (the final age on the charts) on pediatric growth charts [2, 34]. Although regression toward the mean may affect the accuracy of this tool for children of very short or tall parents, it is still a good predictor to identify children who may be at risk for not meeting their growth potential [36]. The sex-specific equations to use are as follows:

Midparental height calculations:

$$ \mathrm{Boys}:\left[\left(\mathrm{mothe}{\mathrm{r}}^{\prime}\mathrm{s}\ \mathrm{height}+13\ \mathrm{cm}\right)+\mathrm{fathe}{\mathrm{r}}^{\prime}\mathrm{s}\ \mathrm{height}\right]/2 $$

$$ \mathrm{Girls}:\left[\mathrm{mothe}{\mathrm{r}}^{\prime}\mathrm{s}\ \mathrm{height}+\left(\mathrm{fathe}{\mathrm{r}}^{\prime}\mathrm{s}\ \mathrm{height}-13\ \mathrm{cm}\right)\right]/2 $$

#### Prematurity

Adjusting for prematurity is an important consideration when assessing growth. The UK Royal College of Paediatrics and Child Health advises plotting children who were born between 32 and 36 weeks plus 6 days of gestation with an adjusted growth chart until age 1 and children born earlier than 32 weeks until age 2 years [37] to provide a more realistic characterization of growth. Ideally, the premature infant should gradually increase both weight and length centiles, maintaining an appropriate weight for length. Plotting concurrently on a standard growth chart may help identify the child’s progress with catch-up growth, nearing standard growth curves [2, 38]. For an infant who has not reached a term gestational age of 37–40 weeks, special growth charts for prematurity should be used until the child reaches the equivalent of 40 weeks of gestation [39].

#### Expected grams of weight gain by age

In children younger than the age of 2 years, weight gain should be compared with WHO standards for average grams of weight gain per day. Although any time interval can be used, longer intervals (e.g., weeks vs. days) provide a more accurate assessment given normal variation. Given that individual growth varies, these standards are in ranges [40]. Ideally, children with kidney diseases should gain weight at a normal rate (Table 2). However, given that some children with kidney diseases have poor linear growth, weight gain must be evaluated in the context of linear growth to ensure proportionality. While poor linear growth can be a marker of inadequate nutrition, poor growth in a child with CKD may also be the result of other factors (e.g., abnormality of the IGF-GH axis, metabolic acidosis, and excessive sodium losses) and in young children weight should not greatly outpace linear growth resulting in an elevated weight for length [2].

**Table 3:** Parameters and frequency of nutritional assessment in children with CKD stages 3b–5D^#^. Dietetic contacts^†^

	CKD 2–3a	CKD 3b–5	CKD 5D
0–6 months of age	3 months	1 month	1 month
6–12 months of age	3 months	1 month	1 month
Age 1 year and older	1 year	3 months	1 month

Adapted from [2], used with permission; dietetic contact recommendations based on expert opinion

^#^Earlier stages of CKD and other kidney diseases are not addressed in this table, as clinical conditions can vary and physician discretion is required. Further details are addressed in [2]

^†^Contacts include in-person, phone, or secure digital communication

#### Nutrition-focused physical examination

A nutrition-focused physical examination (NFPE) involves inspection and palpation of potential areas of fat or muscle wasting to assess nutritional status. The presence of edema suggests that the measured weight overestimates the child’s euvolemic weight [2]. The use of a NFPE to collect data longitudinally will best help identify children at risk. Assessment for specific micronutrients should be considered when indicated by abnormalities on physical exam. Evaluation of skin, hair, and nails not only helps determine the possible presence of micronutrient deficiencies but also may aid in determining malnutrition risk [42, 43]. While it is well recognized that micronutrient losses are common through dialysis, losses or deficiencies may also occur as a result of the third spacing with nephrotic syndrome, CKD-related inflammation, and declining appetite or poor intake [2] and may mandate laboratory evaluation for specific micronutrients (e.g., vitamins and trace elements). Children receiving parenteral nutrition may additionally have specific micronutrient concerns (e.g., excess fat-soluble vitamins and inadequate water-soluble vitamin intake) that may require attention. However, few children with chronic kidney diseases receive chronic parenteral nutrition, except for a select group of HD patients who receive intradialytic parenteral nutrition (IDPN) [44]. Children who are vegetarian or vegan may need to be assessed more thoroughly for the risk of micronutrient deficiencies and the potential need for supplementation. These diets are often high in antioxidant-based micronutrients, but without careful supplementation of zinc, select B vitamins, and iron, the diet may be deficient in these components [45].

The physical examination can be used as part of a subjective global nutritional assessment (SGNA), which has been validated in a general pediatric population [42, 43] and in adults with CKD [46]; there is limited evidence for the use of the SGNA in children with CKD [47]. The SGNA generates a nutritional risk score using objective data, such as anthropometric measurements and weight changes, subjective data including medical history review (appetite changes, reported GI symptoms, and dietary intake), and a physical examination to detect subcutaneous fat loss, muscle wasting, and clinical signs of micronutrient deficiencies. The nutritional risk score determines the likelihood and degree of malnutrition. Guidance on the use of the NFPE and other potential complementary tools is included in the supplementary materials.

#### Secondary measures that may be considered

Growth parameters are the best validated tools for nutritional assessment of children with kidney diseases. However, additional screening measurements may aid in targeting specific potential deficiencies and facilitate a more comprehensive assessment. There is evidence in the pediatric literature that grip strength [48] and waist-to-height ratio [49, 50] are measures to consider. However, additional experience with these measures in clinical practice settings is necessary before their routine use can be recommended. DEXA is another accurate assessment tool but is expensive and primarily used in research settings [51]. Bioelectric impedance analysis (BIA), while used by some centers and trained clinicians for the serial assessment of fluid balance, is typically not useful in assessing anthropometric status [52]. Mid-upper arm circumference (MUAC) measurement is emerging as a clinical tool in pediatrics, but insufficient information is known about its use in children with kidney disease [53, 54]. Triceps skinfold (TSF) thickness measurement is generally not considered to be accurate in children with CKD, is disliked by children, and is difficult to perform [2, 51]. These tools and other evaluators that have been discussed in the CKD literature, along with the recommended frequency of measurement, are provided in Supplementary Table 5.

Table 3 provides recommendations for the frequency of anthropometric evaluation in children with CKD. In centers where dietitian support is not available, pediatricians or pediatric nephrologists may evaluate these parameters, but we encourage the skills of a pediatric renal dietitian are used whenever possible for a nutrition-focused specialized assessment. The dietetic assessment will include evaluation of anthropometric measurements and any other parameters specific to the child’s needs such as dietary evaluation, laboratory follow-up, or other medical-nutrition issues.

## Dietary assessment

2.1. Dietary assessment should be guided by the severity of kidney disease and nutritional concerns, including abnormal growth parameters, excessive or inadequate dietary intake, poor quality of diet, gastrointestinal symptoms, and abnormal biochemical values (grade D, weak recommendation)
  2.1.1. Assess appetite to guide the need for supplementary feeding if a child is not meeting nutritional goals (grade D, weak recommendation)
3.1. Conduct a prospective minimum 3-day diet history when accurate, comprehensive information regarding dietary intake is needed. Although a diet history is preferred, a retrospective diet recall over a 24-h period, preferably inclusive of more than one 24-h period, may also be acceptable for dietary assessment (grade B, moderate recommendation)

### Evidence and rationale

Table 4 outlines the recommended frequency of dietary intake review, as discussed below. A small study by Coleman et al. [55] in children on PD showed that frequent dietetic contact was required to achieve adequate dietary intake for growth. This is particularly important in infants when growth is predominantly nutrition-dependent. Table 3 gives suggested frequency of dietetic contacts based on expert opinion. More frequent evaluations, possibly in earlier CKD stages, may be necessary if the child is not growing adequately or if there are other concerning nutritional issues.

#### Growth

It is well established that children, especially young children, with poor growth experience poorer outcomes. Poor growth is associated with suboptimal neurocognitive development, the potential for a compromised final adult height and increases in morbidity and mortality [2, 4, 5, 28]. Evaluation of dietary intake is recommended as a first-line assessment before other causes of poor growth are examined [2, 56].

The frequency of dietary assessment is influenced by the factors listed in statement 2.1 and should be considered for the individual child. The frequency of evaluation may also need to increase after a dietary modification is made or a supplement is instituted to determine the effectiveness of that intervention. Table 4 provides recommendations for the frequency of dietary assessment by age and stage of CKD. Whereas a child with a stable clinical picture and a stable dietary intake may need less frequent dietary evaluations, it is incumbent upon the clinician to inquire about any change in the child’s usual intake at every hospital visit. Those who are not growing well, have a decline in kidney function, experience an alteration in appetite, have GI symptoms, make poor-quality diet choices, or exhibit changes in biochemical values affected by diet, warrant more frequent evaluation.

#### Under- and overnutrition

Undernutrition in pediatric CKD often presents as protein energy wasting (PEW). There are many factors that may contribute to undernutrition in children with CKD, including uremia, metabolic acidosis, and GI disorders, as discussed throughout this text [2]. Extensive research on malnutrition in pediatric populations, with PEW as a subtype of malnutrition, has been published by the American Society for Parenteral and Enteral Nutrition and the Academy of Nutrition and Dietetics [57–59]. There is also guidance on evaluation of PEW, also referred to as uremic failure to thrive or cachexia, in the pediatric predialysis population. Abraham et al. [26] as part of the Chronic Kidney Disease in Children (CKiD) study, outlined diagnostic criteria for PEW specific for pediatrics, adapted from adult guidelines [60]. Inadequate linear growth, BMI, or MUAC < 5% for height age and BMI or MUAC change of 10% or more from the first to second visits in nonobese children were designated as pediatric-specific criteria by CKiD. Contrary to characteristics in the adult population, low serum cholesterol and transthyretin were rarely seen in the children [26]. It is important to recognize that PEW in children with CKD is associated with increased risk of hospitalization or ER visits and poorer quality of life. It is speculated that as in adults, the inflammation associated with PEW is a cardiovascular risk factor as well. Mortality is increased in adults with CKD and PEW, but such a link has not been established in pediatrics, possibly in part the result of a paucity of data [26]. Evaluation and treatment of PEW are challenging. Management is particularly challenging as energy and protein intake must be adequate, but not excessive, so that fat mass accumulation is not encouraged [61–63].

An abnormal hormonal milieu, as reflected by leptin and ghrelin imbalances, is thought to play a role in the development of PEW in patients with CKD, but the contribution of these factors is not fully understood [61]. While there is a paucity of research on this topic for children on dialysis, a Brazilian study demonstrated that over 40% of children receiving chronic dialysis had mild malnutrition [23]. Children under the age of 5 years are most likely to be underweight and should have a thorough evaluation of energy and protein needs given their vulnerable stage of growth, development, and cognitive gain.

Obesity and overweight are also increasing in pediatric CKD populations, and the adequacy of energy and protein intake must be balanced with the risks of overweight and obesity in relation to cardiovascular, psychosocial, and transplant outcomes [4]. Interventions to reduce the risks of excessive energy intake in this setting should be considered while maintaining nutritional adequacy [2].

#### Quality of diet

Data from the CKiD study has revealed that children with CKD consume diets high in “empty calorie” foods, such as fast foods and chips (crisps) and other snack foods. Even though these children were consuming appropriate ranges of macronutrients, on average, food choices that comprised those macronutrients were poor [64, 65]. Despite adequate or excessive energy (caloric) intake, children may be consuming poor-quality diets with limited essential micronutrients which may require intervention. Age-appropriate dietary intake in terms of quality and quantity, as well as guidance regarding solid food introduction and advancement, is important and is discussed in the *Energy and Protein Requirements for children with CKD stages 2-5 and on dialysis – clinical practice recommendations from the Pediatric Renal Nutrition Taskforce * [66].

#### Gastrointestinal symptoms

Gastrointestinal symptoms are common in pediatric CKD [67] and may include abdominal fullness, vomiting, gastro-esophageal reflux, diarrhea, or constipation. As dietary intake can be altered by such findings, the presence of GI symptoms should prompt a thorough dietary assessment and possible GI consultation.

#### Biochemical values

Dietary evaluation may help identify or rule out nutrition-related contributions to abnormal biochemical values, in terms of electrolytes, bone mineral markers, and protein markers, and help direct nutritional changes or pursuit of other medical interventions [2].

#### Appetite

Data derived from children with CKD in the CKiD study has suggested that appetite can serve as a valuable parameter to monitor because of its correlation with patient outcomes. When the appetite of children was characterized as “very good,” “good,” “fair,” “poor,” or “very poor,” the data revealed an increased risk for hospitalization and emergency room visits in children with a self-reported appetite characterized as anything other than “very good.” A poorer appetite was also associated with a lower quality of life rating [68]. There was an increased incidence of reported poor appetite in children under the age of 5 compared with older children, possibly indicating a greater nutritional risk in the younger cohort [68, 69]. In prior pediatric and adult studies, decreased appetite has also been associated with decreased health-related quality of life [70–72]. The use of the same appetite scale in adults on dialysis (“very good” through “very poor”) also yielded an association between poor appetite and increased inflammatory markers, increased hospitalization, and 4.5–5 times increased mortality risk [70]. Hence, appetite may be an important surrogate marker for nutritional status or overall well-being. Enquiring about appetite at each clinical visit is, in turn, a simple and important step in determining risk. Appetite may also be influenced by abnormal hormone levels (e.g., leptin and ghrelin), as noted in the discussion of PEW [61, 62]. Finally and as noted above, GI symptoms are common in pediatric CKD [67] and can affect both appetite and dietary intake.

#### Dietary data collection

Dietary intake is the most important determinant of the nutritional status of children with kidney diseases [73]. Tools to assess dietary intake have not been validated in children with kidney diseases but have been validated in general pediatric populations [74–78]. There are pros and cons to each tool [79, 80]. A prospective three or more day diet history of food intake is considered the most accurate means to determine dietary intake and is the preferred approach. Multiple 24-h diet recalls of food intake are considered the next most reliable approach to take, with food frequency questionnaires less accurate than recalls or diet histories [81, 82].

Whereas the diet history is the preferred approach, the accuracy of this determination diminishes in older children or in those who have a large dietary intake. The accuracy of this approach may also be compromised in families with limited time and support, presumably due to less eating at home, more data to track, literacy issues, and a plethora of complex reasons [74–76]. Parental involvement in the collection of the dietary data with older children and adolescents can be helpful but may be unrealistic. Although longer periods of recording intake (e.g., 5–7 days) are theoretically the most accurate means to assess intake given the day to day variability in food choices, the time burden may decrease accuracy, often due to underreporting [83]. As noted previously, multiple diet recalls should be considered for patients and families for whom a prospective 3-day diet history is impractical. The clinician must, in turn, evaluate the social dynamics when determining the best method for dietary intake recording for the individual subject and family. In fact, a “usual” or “typical” intake may be the best that can be obtained and should be sought if the alternative is obtaining no dietary intake information. Tools for assessing dietary intake are summarized in Supplemental Table 3 in *The dietary management of calcium and phosphate in children with CKD stages 2-5 and on dialysis – clinical practice recommendation from the Pediatric Renal Nutrition Taskforce* [1].

Technology, such as e-mailed records or food tracking apps, digital assessment, and pictorial food records, may improve accuracy with appropriate patients and families. The use of technology may also help engage families and especially young people in this process [84]. Although there may still be limitations (e.g., forgetting to document a meal or snack), the use of pictures for food recording, especially through smartphone technology, may increase the accuracy of reporting portion sizes and types of foods consumed during prospective record keeping [85].

#### Nutritional impact of dialysis

Specific to patients on PD, the dialysate may provide some intradialytic calories through the absorption of dextrose across the peritoneal membrane. While these calories are rarely accounted for given the below normal intakes of many children receiving PD, this source of calories should not be ignored in the patient being dialyzed with a hypertonic peritoneal dialysis solution, receiving enteral feedings and demonstrating obesity [2]. Amino acid-based solutions may be used in children on PD, but there is minimal evidence for any significant improvement in the nutritional status of children using these dialysis solutions [86]. Protein and other nutrients such as magnesium, calcium, and potassium are passed across the peritoneal membrane through the process of diffusion, and replacement needs to be considered on an individualized basis. High- or low-calcium dialysate solutions are sometimes used to adjust for the amount of calcium lost or absorbed in the process of dialysis. Standard dietary protein additions of 0.15–0.3 g/kg/day are recommended in addition to the SDI to account for protein losses. It should be noted that peritoneal transport status impacts the actual amount of protein and other nutrients that are lost across the peritoneal membrane. Evaluation of transport status through a peritoneal equilibration test (PET), as well as monitoring and potentially quantifying such losses, can help with this assessment and patient management [2, 66, 87, 88].

## Biochemical assessment

3.1. Calculate the normalized protein catabolic rate (nPCR) on a regular basis in adolescent patients on hemodialysis. Utilize individual values and trends to evaluate dietary protein adequacy (grade C, weak recommendation)
4.1. Only consider utilizing serum albumin as a measure of nutritional status after all nonnutritional causes of hypoalbuminemia have been excluded (grade A, strong recommendation)

### Evidence and rationale

#### nPCR

The measurement of multiple biochemical markers may aid in the assessment of the nutritional status of children with kidney disease [2]. However, to date, the only validated biochemical tool to assist in the nutritional assessment of the pediatric CKD population is the nPCR, which has been validated for use in adolescent patients (ages 13–19 years) receiving chronic HD [89–91]. nPCR values may be useful for trending in other ages but are challenging to use given variable rates of growth and related protein turnover in younger children. There is evidence that an nPCR of 1 or greater is indicative of adequate dietary protein intake in adolescent HD patients [89–91]. Younger children who are growing normally have been shown to have values well above 1. Thus, following nPCR trends for individual children can provide information pertaining to weight gain and dietary protein intake once a baseline is established [89]. The following formula is used to calculate nPCR (variables defined in Table 5) and can be standardized in an Excel spreadsheet or similar program:

$$ \mathrm{nPCR}=5.43\times \mathrm{estG}/\mathrm{V}1+0.17 $$

**Table 5:** Variables used for calculating nPCR

Variable	Definition
*G* (mg/min)	[(C2 × V2) – (C1 × V1)]/*T*
C1	Postdialysis BUN (mg/dL)
C2	Predialysis BUN (mg/dL)
V1 (for *G* calculations)	Postdialysis total body water (dL)
V2	Predialysis total body water (dL)
*T*	Time from the end of dialysis treatment to the beginning of the next treatment in minutes
V1 (for nPCR calculations)	Total body water (L)
Volume calculations for *V* values	5.8 dL/kg and pre- or postdialysis weight in kg; for V1 in nPCR calculations: 0.58 and weight in kg

Adapted from [2], used with permission

#### Serum albumin

Although serum albumin has been used as a marker in multiple studies addressing the assessment of nutritional status in pediatric CKD, including several studies contained in the evidence tables [92–95], it lacks sensitivity and specificity as a nutritional parameter [51, 89, 96–98]. The serum albumin is often influenced by nonnutritional factors, including inflammation, infection, and fluid overload [21, 90, 98]. It is a marker of mortality risk and hospitalization, but those outcomes are typically associated with multiple factors besides nutrition [51, 89, 99].

Biochemical markers including sUrea and BUN are sometimes used by clinicians in trending and evaluating protein status [51, 73, 99, 100]; however, research regarding the value of these measures as part of the nutritional assessment is very limited. Furthermore, protein requirements, growth, body size, and urine output all influence these laboratory values, and therefore, clinical judgment is necessary. It is however expected that, in the absence of normal kidney function, children would not have “normal” (i.e., standard age and gender) sUrea or BUN values if dietary protein intake is adequate as failing kidneys are not able to remove nitrogenous waste as in a child with healthy kidneys. Protein nitrogen appearance (PNA) is difficult to determine as a nutritional marker in pediatric PD patients and is also not recommended [2, 51].

Other biochemical markers that may be considered for the nutritional assessment, but which have not been validated for use in children with CKD, are included in the supplementary material.

## Results of the Delphi survey

There were 52 responses to the electronic document with joint responses submitted by some dietitians and physicians from the same facility. All professionals who completed the survey are listed in “Participants in the Delphi survey.”

The 22 clinical practice recommendation statements received an overall 86% consensus with a “strongly agree” or “agree” response and 11% with a “neutral” response. Three statements did not reach the stipulated 70% level of consensus. There were a number of “neutral” responses, largely due to variations in personal practice rather than based on published studies. The taskforce members reviewed the comments and agreed that these statements do not require changing as the GRADE reflects the low level of evidence and indicates that these statements are based on expert opinion.

Seventeen statements received “disagree” ratings, with a median of 1 (range 1–3) rating, and four statements received one “strongly disagree” rating each. The highest level of disagreement was for statement 2.1.1 on assessing appetite, with a 10% “disagree” rate. Respondents were concerned with the subjective nature of the question, and while this clearly remains a subjective assessment, the rating scale used in research studies and its correlation with outcomes have been included. The second highest level of dissent was for statement 1.3.3 on the use of height age for determining BMI *z*-scores (3 “disagree” and 1 “strongly disagree”). On a careful review, the taskforce members confirmed that the use of height age is adequately supported by the literature and that using the 3rd centile as a cut-off was appropriate. Slight modifications were made to six of the statements, and further information was provided under “Evidence and rationale” based on the Delphi feedback.

## Summary of recommendations

A summary of recommendations is provided in Table 6.

**Table 6:** Summary of recommendations

Recommendations	Grade
1. Anthropometric assessment	
1.1 Measure weight, height, or length and the head circumference in children with kidney diseases	A, strong recommendation
1.1.1 Use euvolemic (dry) weight for nutritional assessment, with adjustment of measured weight when indicated (e.g., being on dialysis and having nephrotic syndrome)	A, strong recommendation
1.1.2 Measure recumbent length under 2 years of age and standing height thereafter. When young children are unable to stand for an accurate height measurement, recumbent length can be measured	A, strong recommendation
1.1.2.1 Use a surrogate measurement of height for older children who are unable to stand	D, weak recommendation
1.1.3 Measure the head circumference in all children up to 2 years of age or up to 3 years of age when appropriate centile charts are available	A, strong recommendation
1.2 Plot anthropometric measurements serially on centile growth charts. Use the World Health Organization (WHO) growth chart for all ages or country-specific growth charts, if available, beyond 2 years of age	A, strong recommendation
1.2.1 Calculate *z*-scores (standard deviation scores (SDS)) to complement growth chart plots	X, strong recommendation
1.2.2 Calculate height/length velocity *z*-scores over a minimum period of six months	B, moderate recommendation
1.2.3 Use disorder- or genetic condition-specific growth charts when applicable	B, moderate recommendation
1.2.4 Utilize trends in growth parameters to assist in clinical decision-making	D, weak recommendation
1.3 Calculate the body mass index (BMI) in children aged 2 years and older and weight for length in children younger than age 2	A, strong recommendation
1.3.1 Plot BMI or weight for length on centile growth charts	B; moderate recommendation
1.3.2 Calculate BMI or weight-for-length *z*-scores/SDS to complement growth chart plots	B, moderate recommendation
1.3.3 Use height age for determining the BMI *z*-score/SDS if the child is shorter than the third centile curve on the growth chart, provided the child has not reached their adult height	B, moderate recommendation
1.4 Calculate midparental height and plot the value as a centile to estimate growth potential	C, weak recommendation
1.5 For premature infants, plot weight, length, and weight for length for both gestational and chronological age for the first year of life if born from 32 up to 37 weeks of gestation and through 2 years of age if born prior to 32 weeks of gestation	D, weak recommendation
1.6 Monitor growth parameters routinely in children with kidney diseases, with increased frequency in younger children, and in those children with advanced CKD, with comorbidities, and with risk factors for poor growth and those not meeting nutritional and growth targets	D, weak recommendation
2. Dietary assessment	
2.1 Dietary assessment should be guided by severity of kidney disease and nutritional concerns, including abnormal growth parameters, excessive or inadequate dietary intake, poor quality of diet, gastrointestinal symptoms, and abnormal biochemical values	D, weak recommendation
2.1.1 Assess appetite to guide the need for supplementary feeding if a child is not meeting nutritional goals	D, weak recommendation
2.2 Conduct a prospective minimum 3-day diet history when accurate, comprehensive information regarding dietary intake is needed. Although a diet history is preferred, a retrospective diet recall over a 24-h period, preferably inclusive of more than one 24-h period, may also be acceptable for dietary assessment	B, moderate recommendation
3. Biochemical assessment	
3.1 Calculate the normalized protein catabolic rate (nPCR) on a regular basis in adolescent patients on hemodialysis. Utilize individual values and trends to evaluate dietary protein adequacy	C, weak recommendation
3.2 Only consider utilizing serum albumin as a measure of nutritional status after all nonnutritional causes of hypoalbuminemia have been excluded	A, strong recommendation

### Research recommendations

1. MUAC and nutrition-focused physical exam (NFPE) are validated tools for anthropometric assessment in the general pediatric population. In children with kidney diseases, studies are needed to evaluate the ability of MUAC and NFPE to improve the sensitivity and specificity of methods used to identify children with nutritional deficiencies.
2. Elevated waist-height ratio (WHr) has been shown to correlate with poor outcomes, including increased cardiovascular risk and mortality in children with CKD. Grip strength has also been shown to correlate with malnutrition risk. Studies should evaluate the practicality of measuring WHr and grip strength in children with kidney diseases in the clinical setting and determine the correlation between WHr, measures of cardiovascular risk (e.g., carotid intima–media thickness), and mortality.
3. Air displacement plethysmography (ADP) has been used in small studies of children with kidney diseases to identify malnutrition and determine body composition. Additional studies of ADP should be conducted in children with kidney diseases to determine the ability of this technique to improve the sensitivity and specificity of methods used to identify children with nutritional deficiencies.
4. Blood urea nitrogen (BUN) has been postulated to be an indicator of the adequacy of dietary protein intake. However, BUN also varies based on a variety of other parameters, including GFR, hydration status, and level of catabolism. A 24-h urine collection measured for urea may address some of these limitations but is more burdensome and may be limited by inaccurate collections. Hence, the ability of BUN, 24-h urea collection, or a combination of both should be compared with current evaluation methods in terms of their ability to identify children with CKD who require nutritional intervention to increase protein intake.
5. In adolescents receiving hemodialysis, nPCR has been validated as a technique to identify children who require nutritional intervention. Studies should be conducted in preadolescent patients receiving hemodialysis to determine if nPCR is superior to the standard of care at identifying patients who require nutritional intervention. These studies should explore whether a low nPCR is superior or complementary to current methods used to identify at-risk children receiving hemodialysis.
6. The sensitivity and specificity of serum cholesterol and transthyretin to identify PEW in children receiving dialysis compared with current methods should be determined.
7. The 3-day diet history is the current gold standard for the clinical assessment of nutritional intake in children with CKD. The ability of novel approaches, including food apps, e-mailed records, and pictorial food records, to improve the assessment of dietary intake compared with the 3-day diet history should be studied in children with kidney diseases.
8. Studies should be conducted to evaluate the optimum frequency of nutritional assessments and subsequent interventions in children with kidney diseases. Factors to be considered in the analysis should include patient age, baseline nutritional status, and, if applicable, CKD stage.

## Supplementary information

ESM 1 (DOCX 350 kb).

ESM 2 (DOCX 18 kb).

## Glossary of terms

Arm span: span from the tip of the third finger on the one hand across the body to the third finger on the other hand
Demi-span: span from the center of the chest to the tip of the third finger
Euvolemic: weight without additional or inadequate body fluid
Food recall: verbal collection of past food intake
Food record: prospective record of food intake over 24–72 h or longer
IDPN (intradialytic parenteral nutrition): nutrition given via the hemodialysis access during a hemodialysis session
Length: term used for recumbent linear measurement
Length or height for age: length or height in relation to age norms
MUAC: measurement of the arm circumference in between the elbow and shoulder
NFPE: physical evaluation of a patient to determine risk for malnutrition and potential micronutrient, protein, or fluid concerns
nPCR: a calculation of protein nitrogen appearance in patients on dialysis and used to assess dietary protein intake
PNA (protein nitrogen appearance): protein catabolism calculation, reflecting dietary protein intake
SDS (standard deviation score): amount of positive or negative distance from the mean
PEW: a term for malnutrition specific to muscle losses related to specific medical conditions
TSF (triceps skinfold): amount of fatty tissue that can be pulled away from the muscle of the upper arm
Ulna length: length from the olecranon process to the scaphoid process used to estimate linear height via use of standard equations
Weight for length: the ratio of weight to linear growth in young children
Weight for age: the ratio of weight in relation to age norms
WHr: the numerical ratio of the waist circumference to linear height
*z*-score: distance from the mean
