# Supplementary material for: Assessment of nutritional status in children with kidney diseases—clinical practice recommendations from the Pediatric Renal Nutrition Taskforce
Source: Pediatr Nephrol. 2020 Dec 14;36(4):995–1010. doi: 10.1007/s00467-020-04852-5 (PMC7910229; doi:10.1007/s00467-020-04852-5)
Supplement: Supplementary file 1 — (DOCX 350 kb). [file 467_2020_4852_MOESM1_ESM.docx]

**Assessment of the nutritional status in children with kidney diseases- clinical practice recommendations from the Pediatric Renal Nutrition Taskforce**

**Supplementary Material**

Supplementary Table 1. Search terms strategy used for literature review

| General Terms | Anthropometric Terms | Dietary Assessment Terms | Biochemical Terms |
| --- | --- | --- | --- |
| Pediatric/paediatric  Children  Adolescents  Infants  Chronic Kidney Disease  End-stage kidney disease  Renal  HaemoDialysis  Peritoneal dialysis  Transplant  Nutrition  Assessment  Registries: North American Pediatric Renal Trials and Collaborative Studies (NAPRTCS), US Renal Data System (USRDS), International Pediatric Peritoneal Dialysis Network (IPPN), EDTA | Anthropometric/Anthropometry  Growth  (Dry or Euvolemic) Weight  Height  Length  Puberty  Final (adult) height  Body mass index  Head circumference  Mid upper arm circumference  Growth charts  Bioimpedance  Subjective global (nutritional) assessment  Physical exam  Body composition  Muscle wasting  DXA  IBW  Skinfold thickness/ triceps thickness  Malnutrition  Protein energy wasting  Obesity  SDS or z-scores | Nutrition/nutritional  Dietary (assessment)  Diet/food recall  Food record/diary  Intake  Diet  Appetite | Sodium wasting  Albumin  Prealbumin  nPCR  BUN  Protein losses (in dialysate and urine)  Dialysis adequacy  Fluid allowance  Anemia  Sodium  Potassium, Cholesterol  c-reactive protein, Transferrin  Total protein Creatinine  Acidosis  Leptin  Ghrelin Cytokines  Trace elements (zinc, copper, selenium)  Vitamins |

Supplementary Material Table 2. American Academy of Pediatrics grading matrix
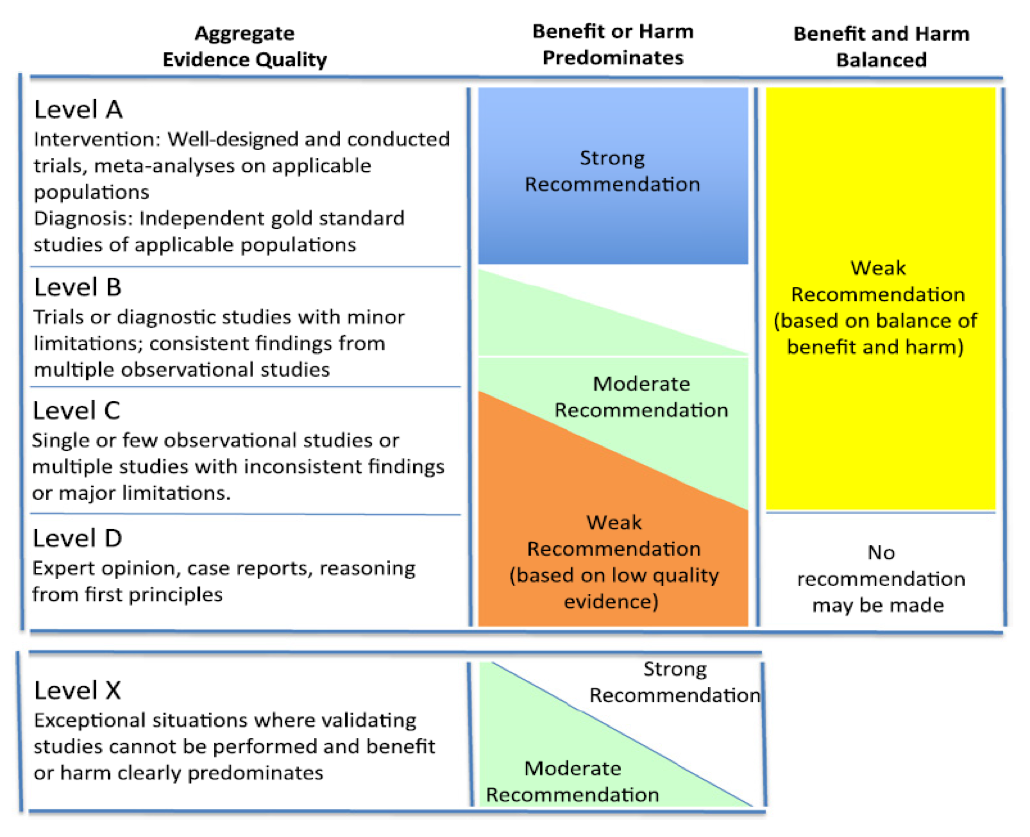


Determining arm span or ulnar length as surrogate measures [1-3]

Arm span: Measure from tip of the middle finger with a non-stretchy tape measure, flat against the body to the tip of the third finger on the other hand to determine estimated height. For demi-span, start at midline of the body and measure to the tip of the third finger and double. Measure twice for greatest accuracy.

Ulnar length:

Males: Height (cm) = 4.605U + 1.308A + 28.003

Females: Height (cm) = 4.459U + 1.315A + 31.485

A = age, U = ulna length

Secondary anthropometric tools to consider

There is limited to moderate evidence for the following measurements. They may be used to refine clinical assessment after performing the primary anthropometric measurements recommended in the guideline statements. They are listed in order of research strength and practicality.

*Grip strength*

Hand grip strength, used as a surrogate for muscle stores and malnutrition risk in adult patients [4-7] and evaluated in a small study in children with CKD [8], has very recently been evaluated in 411 children in the CKiD study. Decreased GFR is associated with reduced muscle function in this population and has also been associated with less exercise [9]. More studies are needed in clinical practice to determine the usefulness and practicality of grip strength as a regular assessment tool.

*Waist to height ratio*

Use of waist circumference measurements have been shown to provide little additional benefit beyond BMI for adiposity or thinness assessment [10]. However, new literature shows that waist to height ratio (WHr) in children with CKD and kidney transplant may be a more sensitive and specific means to predict subclinical cardiovascular changes and the risk of cardiovascular disease, and thus the benefit of measuring fatness or thinness [11, 12]. A WHr of >0.49 predicts overweight status in these populations [11].  The discussion of WHr in this context is as an anthropometric measurement, which has been validated in terms of its potential impact on cardiovascular outcomes. WHr as a component of the assessment for cardiovascular disease is outside of the context of this guideline. Further evaluation of this tool as part of regular clinical practice should be evaluated.

*Mid-upper arm circumference (MUAC)*

MUAC has been validated as a tool to help assess for malnutrition in the general population [13, 14], and recently values have been calculated to assess MUAC z-scores [15] in all children based on United States Centers for Disease Control data [16] to provide normative data and permit the calculation of MUAC z scores for clinical practice.  MUAC has also been used in several studies evaluating the nutritional status of children with kidney diseases [17, 18, 19-21]. Although MUAC has not been independently evaluated in pediatric kidney diseases, a study at a large center included pediatric nephrology patients and determined that there was a high specificity with MUAC and malnutrition [22]. Overall, there is mounting evidence for usefulness in evaluation of overweight and obesity and especially malnutrition in general pediatric populations may make it a helpful addition as a secondary assessment tool in pediatric kidney populations, especially serially or for trending purposes. When measuring MUAC, patients should be at their euvolemic weight to avoid skewing results. A large scale study should be considered to evaluate MUAC as an independent predictor of nutrition status.

*Bioimpedance analysis (BIA)*

The usefulness of BIA or bioimpedance spectroscopy BIS) in helping to assess the nutritional status of pediatric CKD patients [16, 18, 23, 24], is conflicting [25, 26].  If it is used for nutritional assessment, care should be taken to ensure the patient is at euvolemic weight. Current literature suggests that if BIA is used as a nutritional marker or for the determination of euvolemic weight, it should be used serially by a trained clinician with expertise in the determination and use of the measure [27-30].

*Other tools considered*

Also included in the tables, Air Displacement Plethysmography, a tool used in adults and children in the general population [31, 32], has been utilized in a recent, small study of pediatric hemodialysis patients for body composition analysis [20].  However, there is also not yet enough evidence to recommend this tool.

Novel and local assessment tools such as the Pediatric Digital Scaled Malnutrition Risk screening (PeDiSMART), BIA-derived indices, anthropometry-BIA nutrition (ABN) score) have not been validated in larger populations [19, 24, 33].

Skinfold thickness (SFT), total body potassium counting, in vivo neutron activation analysis and isotope dilution have been previously evaluated and determined to have flaws that make them inappropriate for use in children with kidney diseases, while Dual-Energy X-ray Absorptiometry (DEXA) is considered only practical for research [34].

The table below of secondary tools to aid in nutritional assessment represent expert consensus from the PRNT regarding the frequency of usage by stage of CKD and age, if they are being used. However, as some of these tools are only available in select centers or are to be used only by the trained clinicians, using standard operating procedures, these tools are not recommended standardly. They may be useful to complement and refine the primary evaluation with standard anthropometric tools. Clinical judgment is the primary determining factor in their use. Evaluation of rate of weight gain and physical examination are strongly recommended. While tools such as rate of weight gain, physical examination, MUAC, and WHr are inexpensive and relatively simple to use, other measurements may require special equipment or add significant cost to the assessment.

Supplementary Table 3: Parameters for use of secondary tools in nutritional assessment of children with CKD 2-5D

| Measurement | Frequency | Stages of CKD | Age Parameters | Comments for usage |
| --- | --- | --- | --- | --- |
| Expected grams of weight gain by age | Monthly or at each clinic visit | All | ≤2 years of age | Any length of time can be evaluated; greater periods of time reflect overall growth trend; strongly recommended as supplement to growth charts |
| Physical examination | As needed per clinical judgment | All | All | Serial evaluation valuable to assess changes; may be influenced by PD fluid |
| Waist to height ratio | 6-12 months | CKD 2-5 | ≥4 years of age | Can be used concurrently with BMI; may better reflect body composition |
| Mid-upper arm circumference | 6 months | All | See WHO guidelines by age | Marker of malnutrition |
| Hand grip strength | 6 months | All | ≥6 years of age | Marker of malnutrition, musculature; further research needed before standard use can be recommended |
| Bioimpedance analysis | As needed per clinical judgement | Dialysis only | ≥1 year of age | Procedure to be conducted serially and only by trained personnel |

Recommendations influenced by the following references: 4, 8, 9, 11, 12, 17, 20-22, 28-30, 36 37

Additional biochemical measurements to consider

Serum cholesterol and transthyretin, in addition to serum albumin, have been used for validation of protein energy wasting (PEW) in adult populations [38].  However, low cholesterol and transthyretin were not shown to be common in PEW in pediatric populations [39]. Serum bicarbonate and anemia [40] are associated with poor growth in children with CKD [41-43], and although associated with poor nutritional status [44], it is not an independent tool for evaluation of nutrition.  Although depressed serum creatinine can be a marker of poor nutritional status [23] in the general population, it is difficult to be used as part of the nutritional assessment in CKD since an elevated serum creatinine level is expected. In adult dialysis patients, use of the urea to creatinine ratio has been used to assess malnutrition [45], but this has not been evaluated in pediatrics. Transthyretin (prealbumin) is not typically assessed in CKD because accumulation of serum retinol artificially elevates prealbumin levels [46]. However, the finding of a low prealbumin despite the likelihood of artificial elevation, can be indicative of an increased risk of malnutrition. It may be indicative of fluid status and is often used in research settings. However, transthyretin is not commonly measured because it is a more expensive test and may be skewed upward compared to albumin [47]. Retinol binding protein (RPB) is sometimes considered a nutritional marker, however in CKD it is altered as kidney function declines. Also, RBP is often skewed as early as stage 2 CKD. Thus it is not an ideal choice for this population [48, 49]. These biochemical indices, although not valuable as independent markers, can be assessed and evaluated in tandem with other assessment tools to provide further evaluative scope for nutrition needs in CKD.

Adipokines, leptin, CRP, low phosphorus with poor intake, and other inflammatory markers are emerging as a potential tools and may be useful in the assessment of nutritional status [50, 51], but inadequate data is available at this time.  Apelin was recently shown to have no relationship to assessment of malnutrition in pediatric CKD [52]. Inflammatory markers have been shown to be relevant in adult dialysis patients, but the association of inflammation and malnutrition in pediatrics has been less clear-cut [21, 41, 43]. Evaluation of anemia management through laboratory measurements, such as serum iron and ferritin are outside the scope of this document. There are some weak links between anemia markers and malnutrition in adult CKD patients, but there are no studies in pediatric CKD [53].

References

1. Yabanci N, Kilic S, Simsek II (2010) The relationship between height and arm span, mid-upper arm and waist circumference in children. Ann Hum Biol 37(1):70-5
2. Bell KL, Davies PSW (2006) Prediction of height from knee height in children with cerebral palsy and non-disabled children. Ann Hum Biol 33(4):493-9
3. Haapala H, Peterson MD, Daunter A, Hurvitz EA (2016) Agreement between actual height and estimated height using segmental limb lengths for individuals with cerebral palsy. Am J Phys Rehabil 94(7):539-46
4. Heimburger O, Qureshi AR, Blaner WS, Berglund L, Stenvinkel P (2000) Hand-grip muscle strength, lean body mass, and plasma proteins as markers of nutritional status in patients with chronic renal failure close to start of dialysis therapy. Am J Kidney Dis 35:1213-25
5. Wang AY, Sea MM, Ho ZS. Lui SF, Li PK, Woo J (2005) Evaluation of hand-grip strength as a nutritional marker and prognostic indicator in peritoneal dialysis patients. Am J Clin Nutr 81:79-86
6. Pinto AP, Ramos CI, Meireles MS, Kamimura MA, Cuppari L (2015) Impact of hemodialysis session on handgrip strength. J Bras Nefrol 37:451-57
7. Pagels A, Heiwe S, Hylander B (2006) Nutritional status and handgrip strength in pre-dialysis patients. J Ren Care 32:151-5
8. Bakr AMAEB, Hasaneen BM, Bassiouni DARH (2018) Assessment of nutritional status in children with CKD using hand grip strength tool. J Ren Nutr 28(4):265-9
9. Hogan J, Schneider MF, Pai R, Denburg MR, Kogon A, Brooks ER, Kaskel FJ, Reidy KJ, Saland JM, Warady BA, Furth SL, Greenbaum LA (2020) Grip strength in children with chronic kidney disease. Pediatr Nephrol 35(5):891-899
10. Patel HP, Saland JM, Ng DK, Jiang S, Warady BA, Furth SL, Flynn JT (2017) Waist circumference and body mass index in children with chronic kidney disease and metabolic, cardiovascular and renal outcomes. J Pediatr 191:133-9
11. Sgambat K, Roem J, Mitsnefes M, Portale AA, Furth S, Warady BA Moudgil A (2018) Waist-to-height ratio, body mass index, and cardiovascular risk profile in children with chronic kidney disease. Pediatr Nephrol 33(9):1577-1583
12. Sgambat K, Clauss S, Moudgil A (2018) Comparison of BMI, waist circumference, and waist-to-height ratio for identification of subclinical cardiovascular risk in pediatric kidney transplant recipients. Pediatr Transplant 1:1-8
13. Addo OY, Himes JH, Zemel BS (2017) Reference ranges for midupper arm circumference, upper arm muscle area, and upper arm fat area in US children and adolescents aged 1–20 y. Am J Clin. Nutr 105(1):111–120
14. Modi P, Nasrin S, Hawes M, Glavis-Bloom J, Alam NH, Hossain MI, Levine AC (2015) Midupper Arm Circumference Outperforms Weight-Based Measures of Nutritional Status in Children with Diarrhea. J Nutr 145(7):1582-7
15. Abdel-Rahman SM, Bi C, Thaete K (2017) Construction of Lambda, Mu, Sigma values for determining mid-upper arm circumference z scores in U.S. children aged 2 months through 18 years. Nutr Clin Pract 32:68-76
16. Centers for Disease Control and Prevention (2018) National Health and Nutrition Examination Survey: Survey and Data Collection Systems. https://www.cdc.gov/nchs/surveys.htm, accessed October 31, 2018
17. Garcia De Alba Verduzco J, Fabiola Hurtado Lopez E, Ponton Vazquez C, de la Torre Serrano A, Romero Velarde E, Manuel Vasquez Garibay E (2018) Factors associated with anthropometric indicators of nutritional status in children with chronic kidney disease undergoing peritoneal dialysis, hemodialysis, and after kidney transplant. J Ren Nutr 28(5):352-358
18. Ponton-Vazquez C, Vasquez-Garibay M, Hurtado-Lopez EF, de la Torre Serrano A, Garcia GP, Romero-Velarde E (2017) Dietary intake, nutritional status, and body composition in children with end-stage kidney disease on hemodialysis or peritoneal dialysis. J Ren Nutr 27(3):207-15
19. Apostolou A, Printza N, Karagiozoglou-Lampoudi T, Dotis J, Papachristou F (2014) Nutrition assessment of children with advanced stages of chronic kidney disease – a single center study. Hippokratia 18(3):212-16
20. Wong-Vega M, Srivaths PR (2017) Air displacement plethysmography versus bioelectrical impedance to determine body composition in pediatric dialysis patients.  J Ren Nutr 27(6):439-444
21. Canpolat N, Caliskan S, Sever L, Tasdemir M, Ekmekci OB, Pehlivan G, Shroff R (2013) Malnutrition and its association with inflammation and vascular disease in children on maintenance dialysis. Pediatr Nephrol 28(11):2149-56
22. Stephens K, Orlick M, Beattie S, Snell A, Munsterman K, Oladitan L, Abdel-Rahman S (2020) Examining Mid-Upper Arm Circumference Malnutrition z-Score Thresholds. Nutr Clin Pract 35(2):344-352
23. Edefonti A, Paglialonga F, Picca M (2006) A prospective multicenter study of nutritional status in children maintained on peritoneal dialysis. Nephrol Dial Transplant 21:1946-51
24. Edefonti A, Picca M, Damiani B, Garavaglia R, Loi S, Ardissino G, Marra G, Ghio L (2001) Prevalence of malnutrition assessed by bioimpedance analysis and anthropometry in children on peritoneal dialysis. Perit Dial Int 21:172-179
25. National Kidney Foundation (2009) KDOQI Clinical Practice Guideline for Nutrition in Children with CKD: 2008 update. Am J Kidney Dis 53(suppl 2):S1-S124
26. Milani GP, Groothoff JW, Vianello FA, Fossali EF, Paglialonga F, Edefonti A, Agostoni C, Consonni D, van Harskamp D, van Goudoever JB, Schierbeek H, Oosterveld MJ (2017) Bioimpedance and fluid status in children and adolescents treated with dialysis. AJKD 69(3):428-35
27. Mastrangelo A, Paglialonga F, Edefonti A (2014) Assessment of nutritional status in children with chronic kidney disease and on dialysis. Pediatr Nephrol 29:1349-58
28. Paglialonga F, Civitillo CF, Groppali E, Edefonti A (2010) Assessment of nutritional status in children with chronic kidney disease. Minerva Pediatr 62:295-306
29. Paglialonga F, Edefonti A (2009) Nutrition assessment and management in children on peritoneal dialysis. Pediatr Nephrol 24:721-30
30. Eng CSY, Bhowruth D, Mayes M, Stronach L, Blaauw M, Barber A, Rees L, Shroff RC (2018) Assessing the hydration status of children with chronic kidney disease and on dialysis: a comparison of techniques. Nephrol Dial Transplant 1;33(5):847-55
31. Fields DA, Goran MI, McCrory MA (2002) Body composition assessment via air-displacement plethysmography in adults and children: a review. Am J Clin Nutr 75:453-67
32. Ittenbach RF, Buison AM, Stallings VA, Zemel BS (2006) Statistical validation of air-displacement plethysmography for body composition assessment in children. Ann Hum Biol 33(2):187-201
33. Edefonti A, Picca M, Paglialonga F, Loi S, Grassi MR, Ardissino G, Marra G, Ghio L, Fossali E (2002) A novel objective nutritional score for children on chronic peritoneal dialysis. Pert Dial Int 22(5):602-7
34. Foster BJ, Leonard MB (2004) Measuring nutritional status in children with chronic kidney disease. Am J Clin Nutr 80:801-14
35. World Health Organization (2018) WHO Child Growth Standards, www.who.int/childgrowth/en, accessed October 23, 2018
36. Secker D, Jeejeebhoy KN (2007) Subjective Global Nutritional Assessment for children. Am J Clin Nutr 85:1083-9
37. Secker D, Jeejeebhoy KN (2012) How to perform Subjective Global Nutritional assessment in children. J Acad Nutr Diet 112(3):424-431
38. Fouque D, Kalantar-Zadeh K, Kopple J, Cano N, Chauveau P, Cuppari L, Franch H, Guarnieri G, Ikizler TA, Kaysen G, Lindholm B, Massy Z, Mitch W, Pineda E, Stenvinkel P, Trevino-Becerra A, Wanner C (2008) A proposed nomenclature and diagnostic criteria for protein-energy wasting in acute and chronic kidney disease. Kidney Int 73:391-9
39. Abraham AG, Mak RH, Mitsnefes MM, White C, Moxey-Mimms M, Warady B, Furth SL (2014) Protein energy wasting in children with CKD. Pediatr Nephrol 29(7):1231-8
40. Salas P., Pinto V., Rodriguez J., Zambrano MJ., Mericq V. Growth Retardation in Children with Kidney Disease. *Int J Endocrinolo.* 2013, ID 970946. doi: 10.1155/2013/970946
41. Foster BJ, Kalkwarf HJ, Shults J, Zemel BS, Wetzsteon RJ, Thayu M, Foerster DL, Leonard MB (2011) Association of chronic kidney disease with muscle deficits in children. J Am Soc Nephrol 22:377-86
42. Rodig NM, McDermott KC, Schneider MF, Hotchkiss HM, Yadin O, Seikaly MG, Furth SL, Warady BA (2014) Growth in children with chronic kidney disease: a report from the chronic kidney disease in children study. Pediatr Nephrol 29(10):1987-95
43. Sylvestre LC, Fonseca KPD, Stinghen AEM (2007) The malnutrition and inflammation axis in pediatric patients with chronic kidney disease.  Pediatr Nephrol 22:864-73
44. Gupta A, Mantan M, Sethi M (2016) Nutritional Assessment in Children with Chronic Kidney Disease. Saudi J Kidney Dis Transpl 27(4):733-9
45. Fassinger N, Imam A, Klurfeld DM (2010) Serum retinol, retinol-binding protein, and transthyretin in children receiving dialysis. J Ren Nutr 20(1):17-22
46. Kalantar-Zadeh K, Cano NJ, Budde K, et al (2011) Diets and enteral supplements for improving outcomes in chronic kidney disease. Nat Rev Nephrol 7(7):369-384
47. Uwaezuoke SN, Ayuk AC, Muoneke VU, Mbanefo NR (2018) Chronic kidney disease in children: Using novel biomarkers as predictors of disease. Saudi J Kidney Dis Transpl 29(4):775-784
48. Manickavasagar B, McArdle AJ, Yadav P, Shaw V, Dixon M, Blomhoff R, O’Connor G, Rees L, Ledermann S, Van’t Hoff W, Shroff R (2015) Hypervitaminosis A is prevalent in children with CKD and contributes to hypercalcemia. Pediatr Nephrol 30(2):317-325
49. Tufan F, Yildiz A, Dogan I, Yildiz D, Sevinir S (2015) Urea to creatinine ratio: a forgotten maker of poor nutritional state in patients undergoing hemodialysis treatment. Aging Male 18(1):49-53
50. Maggio MC, Montaperto D, Maringhini S, Corrado C, Gucciardino E, Corsello G (2014)  Adiponectin, resistin and leptin in paediatric chronic renal failure: correlation with auxological and endocrine profiles. J Nephrol 27(3):275-9
51. Yilmaz D, Sonmez F, Karakas S, Yavascan O, Aksu N, Omurlu IK, Yenisey C (2016) Evaluation of nutritional status in children during predialysis, or treated by peritoneal dialysis or hemodialysis. J Trop Pediatr 62:178-84
52. Yavuz S, Cetinkava S, Anarat A, Bayazit AK (2014)  Apelin and nutritional status in children on dialysis. Ren Fail 36(8):1233-8
53. Aggarwal HK, Jain D, Chauda R, Bhatia S, Sehgal R (2018) Assessment of Malnutrition Inflammation Score in Different Stages of Chronic Kidney Disease. Pril (Makedon Akad Nauk Umet Odd Med Nauki*)* 39(2-3):51-61.

Other references in the evidence tables:

1. Ku E, Glidden DV, Hsu C, Portale AA, Grimes B, Johansen KL (2016) Association of body mass index with patient-centered outcomes in children with ESRD.  J Am Soc Nephrol 27:551-8
2. Silva VR, Soares CB, Magalhaes JO, de Barcelos IP, Cergueira DC, Simoes e Silva AC, Oliveira EA (2015) Anthropometric and biochemical profile of children and adolescents with chronic kidney disease in a pre-dialysis program. Scientific World Journal 2015:810758
3. Fadel F, Elshamaa MF, Essam RG, Elghoroury EA, El-Saeed GS, El-Toukhy SE, Ibrahim MH (2014)  Some amino acids levels : glutamine, glutamate, and homocysteine, in plasma of children with chronic kidney disease. Int J BIomed Sci 10(1):36-42
4. Sahpazova E, Kumanovaska D, Todorovska L, Bogdanovska A (2006) Nutritional status, protein intake and progression of renal failure in children. Pediatr Nephrol 21(12):1879-83
5. Gupta A, Mantan M, Sethi M (2016) Nutritional Assessment in Children with Chronic Kidney Disease. Saudi J Kidney Dis Transpl 27(4):733-9
6. Yilmaz D, Sonmez F, Karakas S, Yavascan O, Aksu N, Omurlu IK, Yenisey C (2016) Evaluation of nutritional status in children during predialysis, or treated by peritoneal dialysis or hemodialysis. J Trop Pediatr 62:178-84
