# Supplementary material for: Assessment of nutritional status in children with kidney diseases—clinical practice recommendations from the Pediatric Renal Nutrition Taskforce
Source: Pediatr Nephrol. 2020 Dec 14;36(4):995–1010. doi: 10.1007/s00467-020-04852-5 (PMC7910229; doi:10.1007/s00467-020-04852-5)
Supplement: Supplementary file 2 — (DOCX 18 kb). [file 467_2020_4852_MOESM2_ESM.docx]

Table 4: Summary of recommendations

| **Recommendations** | **Grade** |
| --- | --- |
| **1. Anthropometric Assessment** | |
| **1.1 Measure weight, height or length, and head circumference in children with kidney diseases.**  **1.1.1 Use euvolemic (dry) weight for nutritional assessment, with adjustment of measured weight when indicated (e.g. being on dialysis, having nephrotic syndrome).**   - - 1. **Measure recumbent length under 2 years of age and standing height thereafter. When young children are unable to stand for an accurate height measurement, recumbent length can be measured.**   **1.1.2.1 Use a surrogate measurement of height for older children who are unable to stand.**  **1.1.3 Measure head circumference in all children up to 2 years of age, or up to 3 years of age when appropriate centile charts are available.** | A; strong recommendation  A; strong recommendation  A; strong recommendation  D; weak recommendation  A, strong recommendation |
| **1.2 Plot anthropometric measurements serially on centile growth charts. Use the World Health Organization (WHO) growth chart for all ages or country-specific growth charts, if available, beyond 2 years of age.**  **1.2.1 Calculate z-scores [standard deviation scores (SDS)] to complement growth chart plots.**   - - 1. **Calculate height/length velocity z-scores over a minimum period of six months.**     2. **Use disorder or genetic condition-specific growth charts when applicable.**      - - 1. **Utilize trends in growth parameters to assist clinical decision-making.** | A; strong recommendation  X; strong recommendation  B; moderate recommendation  B; moderate recommendation  D; weak recommendation |
| **1.3 Calculate body mass index (BMI) in children aged 2 years and older, and weight-for-length in children younger than age 2.**   - - 1. **Plot BMI or weight-for-length on centile growth charts.**     2. **Calculate BMI or weight-for-length z-scores/SDS to complement growth chart plots.**      - - 1. **Use height age for determining BMI z-score/SDS if the child is shorter than the third centile curve on the growth chart, provided the child has not reached their adult height.** | A; strong recommendation  B; moderate recommendation  B; moderate recommendation  B; moderate recommendation |
| **1.4 Calculate mid-parental height and plot the value as a centile to estimate growth potential.** | C; weak recommendation |
| **1.5 For premature infants, plot weight, length and weight-for-length for both gestational and chronological age for the first year of life if born from 32 up to 37 weeks gestation, and through 2 years of age if born prior to 32 weeks gestation**. | D; weak recommendation |
| **1.6 Monitor growth parameters routinely in children with kidney diseases, with increased frequency in younger children, and in those children with advanced CKD, with comorbidities, with risk factors for poor growth and those not meeting nutritional and growth targets.** | D; weak recommendation |
| **2. Dietary Assessment** | |
| **2.1 Dietary assessment should be guided by severity of kidney disease and nutritional concerns, including abnormal growth parameters, excessive or inadequate dietary intake, poor quality of diet, gastrointestinal symptoms and abnormal biochemical values.**   - - 1. **Assess appetite to guide the need for supplementary feeding if a child is not meeting nutritional goals.** | D; weak recommendation  D; weak recommendation |
| **2.2 Conduct a prospective minimum 3-day diet history when accurate, comprehensive information regarding dietary intake is needed. Although a diet history is preferred, a retrospective diet recall over a 24-hour period, preferably inclusive of more than one 24-hour period, may also be acceptable for dietary assessment.** | B; moderate recommendation |
| **3. Biochemical Assessment** | |
| - 1. **Calculate normalized protein catabolic rate (nPCR) on a regular basis in adolescent patients on hemodialysis. Utilize individual values and trends to evaluate dietary protein adequacy.**   **3.2 Only consider utilizing serum albumin as a measure of nutritional status after all non-nutritional causes of hypoalbuminemia have been excluded.** | C; weak recommendation  A; strong recommendation |
